# Effect of Reconstituted Broth on the Taste-Active Metabolites and Sensory Quality of Stewed and Roasted Pork-Hock

**DOI:** 10.3390/foods9040513

**Published:** 2020-04-20

**Authors:** Yi Yang, Daodong Pan, Ying Wang, Jun He, Yi Yue, Qiang Xia, Guanghong Zhou, Jinxuan Cao

**Affiliations:** 1 State Key Laboratory for Managing Biotic and Chemical Threats to the Quality and Safety of Agro-Products, Ningbo University, Ningbo 315211, China; yyforever1993@163.com (Y.Y.); pandaodong@nbu.edu.cn (D.P.); hejun@nbu.edu.cn (J.H.); xiaqiang@nbu.edu.cn (Q.X.); caojinxuan@nbu.edu.cn (J.C.); 2 College of Food Science and Technology, Wuhan Polytechnic University, Wuhan 430023, China; appleme1993@163.com; 3 College of Food Science and Technology, Nanjing Agricultural University, Nanjing 210095, China; guanghong.zhou@hotmail.com

**Keywords:** stewed pork-hock in soy sauce, spray drying treatment, reconstituted broth, metabolomics, sensory evaluation

## Abstract

Stewed pork-hock in soy sauce (SPHSS) is a cuisine that is stewed in broth with abundant taste-active compounds. Broth plays an important role in determining the meat taste. In order to promote the comprehensive utilization of the broth we treated it by spray drying, and secondary processed it into reconstituted broth. Two new products: SPH (stewed pork-hock with reconstituted broth) and MRPH (marinated and roasted pork-hock with reconstituted broth) were processed. Their metabolome consisted of amino acids, sugars, organic acids, nucleic acids and their derivatives. PC1 and PC2 explained a total of 63.07% and 35.31% of the variation, respectively. All the metabolite levels in SPH were higher than those in SPHSS, except for histidine and phosphorylcholine. SPH kept the highest levels of total FAAs and total sugars, which corresponded to the highest score of overall taste in the three products. These results demonstrated that reconstituted broth can promote the metabolite concentration in and improve the taste of pork-hock. Compared with marinating and roasting, reconstituted broth was more suitable for stewing pork-hock. This study preliminarily explored a feasible method to comprehensively utilize the surplus broth in food processing. SPH with a shortened processing time by a reconstituted broth have potential application in the industry due to the high concentrations of taste metabolites.

## 1. Introduction

Chinese cuisine, one of the most sophisticated cuisines in the world, is well received due to its rich flavor and delicious taste. Stewed pork-hock in soy sauce (SPHSS) is an ethnic Chinese cuisine and is very popular in Asia due to its special mouth-feel and taste. Pork-hock is the knuckle part of pig’s leg, which is mainly composed of protein and lipids. The processing method of SPHSS includes four steps: washing, pre-boiling, frying and stewing in broth for a long time [1]. SPHSS presents a strong-sauce flavor due to the addition of spices and seasonings in the broth during stewing. Relish is one of the most important attributes of the meat quality and is associated with molecular metabolites, such as free amino acids (FAAs), small peptides, inorganic salts, sugars and succinic acid [2,3,4,5]. As for stewed meat products, broth contains abundant taste-active metabolites and plays an important role in the taste. Large numbers of FAAs, 5ʹ-nucleotides and minerals have been found migrating from the chicken meat into the broth during stewing [6,7]. The broth of stewed chicken also contains abundant taste-active compounds, such as isoleucine (bitterness), glutamate and IMP (umami), alanine and glycine (sweetness), nucleotides and minerals [8].

A certain amount of the taste-active compounds in the broth permeated and thus enriched the taste of the SPHSS. Currently, the utilization degree of broth in a factory is very low due to it spoiling easily and thus being inconvenient to store. A large amount of spoiled broth was dipped into underground pipelines directly with a high salt content. It caused very serious environmental pollution. How to improve the utilization of broth is a very important program, both in food processing and the environmental protection field. Drying the broth to get powders has been demonstrated to be an effective method. Food powders, including orange juice powders, soymilk powders and mango powders, were applied in producing innovative formulations by using spray drying (SD) treatment [9,10,11,12]. SD treatment is an operation by which a liquid product is atomized in a hot gas current to obtain high-quality powders instantaneously [13]. In the Chinese meat industry, some factories tried to get broth powder by SD treatment in order to facilitate the storage of broth and shorten the stewing time of SPHSS. However, customers are always concerned about the sensory quality of the reconstituted broth products. Up to now, no study has been done to explore the composition and taste of reconstituted broth products. In this research, we aimed (1) to explore a feasible method to comprehensively utilize the surplus broth in food processing, (2) to know the effect of reconstituted broth on the taste-active metabolites of pork-hock, and (3) to create a shortened processing method for pork-hock by using reconstituted broth.

A reconstituted broth was prepared by mixing broth powders and water in a certain proportion. In our study, reconstituted broth was used to process two new products: SPH (stewed pork-hock with the reconstituted broth) and MRPH (marinated and roasted pork-hock with the reconstituted broth). In China, stewing and roasting were the two most commonly used methods for processing pork-hock. This is the reason that we chose the two processing methods for researching the taste effect of reconstituted broth. Finally, the taste sensory quality and taste metabolites of the SPHSS were compared with SPH. Meanwhile, the taste sensory quality and taste metabolites of the SPH and MRPH were also compared; we also determined which processing method is more suitable for reconstituted broth.

## 2. Materials and Methods

### 2.1. Processing of Broth

We purchased 40 pork-hocks (the joint of the swine’s foreleg and pettitoes), which were from a commercial abattoir, with an average weight of approximately 1–1.2 kg from Duroc × Landrance cross-breed pigs. The process method was according to our previous study [1]. The forty pork-hock pieces were chopped, washed, pre-boiled and fried with 21 g/kg bean paste for 10 min, stewed in broth (including 950 mL/kg water, 3 g/kg Chinese prickly ash, 4 g/kg star anise, 4 g/kg cinnamon, 37 g/kg scallion, 23 g/kg ginger, 16 g/kg garlic, 80 g/kg soy sauce, 29 g/kg bean paste, 20 g/kg rice wine and 35 g/kg sugar) for 90 min. The pork-hocks were stewed in a cooking pot that kept the stewing temperature at 80 °C. After stewing, the broth and solid mixture (pork-hocks and spices) were separated and placed at room temperature until cooling. Finally, 25 L broth was obtained and stored at 4 °C before spray drying.

### 2.2. Spray Drying of Broth

Twenty-five liters of broth were heated by an induction cooker and fed into a laboratory-scale spray dryer (model YC-018, Shanghai Yacheng Instrument Equipment Co., Ltd., Shanghai, China) by a peristaltic pump with preheating before using them. The inlet air temperature was set at 150 ± 1 °C and the outlet air temperature was kept at 68 ± 1 °C. The peristaltic pump speed was set at 15 rpm. The compressor air pressure was 0.1 MPa and the processing capacity was 1.5 L/h. After the completion of the experiment and when the inlet air temperature fell below 90 °C, the powders were collected from the collection vessel. In our study, 3.2 kg powder was collected and packed in sealed bags after cooling and kept in a dry environment to prevent moisture adsorption. The proportion of powder and water in the reconstituted broth were mixed at a ratio of 8:95 (g/mL).

### 2.3. Processing of SPHSS, SPH and MRPH

Thirty Duroc × Landrance cross-breed pigs with weights ranging from 80 to 100 kg were selected and slaughtered in a commercial abattoir. Thirty pork-hock pieces (the joint of the swine’s foreleg and pettitoes) with an average weight of approximately 1–1.2 kg were chosen as samples and held for 24 h at 4 °C after slaughter. The 30 pieces of pork-hock were divided into 3 groups (SPHSS, SPH and MRPH; SPH was chosen as the control group). Each group included 10 pieces of pork-hock that were cut into small well-proportioned pieces. SPHSS: 10 pieces of pork-hock were processed; the processing method was same to the processing method of broth. SPH: 10 pieces of pork-hock stewed with reconstituted broth (0.8 kg broth powders and 9.5 L water were mixed and heated until boiled) at 80 °C for 90 min. MRPH: 10 pieces of pork-hock were marinated with reconstituted broth (the same with the reconstituted broth of SPH) at 4 °C for 12 h; then, the marinated pork-hocks were roasted at 220 °C for 40 min. After the preparation of SPHSS, SPH and MRPH, their muscle parts were chosen as samples and wrapped in the silver paper, separately. The samples were stored at −40 °C before metabolite extraction.

### 2.4. Metabolite Extraction

Metabolite extraction was performed according to a previous method [4]. A total of 30 pieces of cooked pork-hock (10 pieces of SPHSS, SPH and MRPH, respectively) were cut into small cubes. For each piece, 100 g of the small cubes were minced by a mincing machine (model JYL-C012, Jiuyang Co., Ltd., Jinan, China). Among the 100 g of each sample, we randomly chose 0.4 g mixed with 1 mL of methanol/water (2:1, *v/v*), 5 ceramic beads (Φ = 3 mm) and 1 g ceramic beads (Φ = 0.05 mm), and then put it into a 2 mL grinding tube (Omni International, Inc., Kennesaw, GA, USA). The mixture was homogenized 5 times at 5000 rpm through a QIAGEN TissueLyser II (Hilden, Germany), centrifuged at 12,400·*g* for 10 min at 4 °C. The above extraction process was performed twice. After combining the extracting solution, the methanol was removed through a CentriVap Concentrator (Labconco, Kansas, MO, USA). The water was removed through a vacuum freeze dryer (model Christ ALPHA 1-2 LD plus, Germany). The farinose solid was obtained and dissolved into 550 μL of phosphate buffer (0.15 M K2HPO4/NaH2PO4, pH 7.55), containing 0.01% NaN3, 50% D2O and 0.001% sodium 3-trimethylsilyl (2, 2, 3, 3-d4), vortex-mixed, and centrifuged at 12,400·*g* and 4 °C for 10 min. A total of 500 μL of supernatant was pipetted into a 5 mm NMR tube (Norell, ST500-7; Norell, Inc., Landisville, NJ, USA) for NMR analysis.

### 2.5. NMR Analysis

The NMR analysis was determined according to previous methods [4]. ^1^H NMR spectra were conducted at 298 K on a Bruker AVIII 600 MHz NMR Spectrometer (Bruker Biospin, Rheinstetten, Germany) combined with an inverse detection probe under the operating condition of 600.13 MHz for ^1^H. To collect the spectra of metabolite profiles of each sample, a standard one-dimensional pulse sequence (RD-90°-t_1_-90°-t_m_-90°-acquisition) was conducted with a mixing time (t_m_, 80 ms) and weak irradiation to suppress the water signal during the recycle delay (RD, 2 s). A 90° pulse length was set to 13.8 μs; the parameter t_1_ was set to 4 μs. A total of 32 transients were collected into 32 k data points with a spectral width of 20 ppm. All free induction decays (FIDs) employed an exponential window function with a 1 Hz line broadening factor prior to Fourier transformation (FT). One of the 30 samples was chosen to test a catalogue of two-dimensional (2D) NMR spectra for specifying the metabolites.

### 2.6. Data Analysis

The data analysis was determined according to the previous methods [4]. ^1^H NMR data were analyzed by TOPSPIN software (Bruker Biospin, Rheinstetten, Germany). The normalized NMR data sets were analyzed by multivariate data analysis (the software package SIMCA-P^+^, version 11.0, Umetrics, Umea, Sweden). Principal component analysis (PCA) was executed using mean-centered scaling; the scores and loading plots expressed the final consequence. To acquire a general view of the variation among groups, each point in the scores represented an individual sample; yet, the loading plots represented the magnitude and manners of the NMR signals for the classification. The orthogonal projection to latent structure with discriminant analysis (OPLS-DA) method with 7-fold cross-validation and unit-variance scaling was performed to further analyze any intrinsic biochemical dissimilarities between the different samples. The whole OPLS-DA models were validated by a cross-validated residuals (CV-ANOVA) approach with *p* < 0.05 as the significance level. The results were also visualized in the scores and coefficient plots. MATLAB (software, version 7.1, MathWorks, Natick, MA, USA) produced the coefficient plots that were color-coded with the absolute values of the correlation coefficients (*r*). Metabolite content data were analyzed by SAS 8.0 software.

### 2.7. Sensory Evaluation

Sensory analysis included six types of taste characteristics (sweetness, umami, bitterness, sourness, saltiness and overall taste). The sensory profiling method was used in our study [14]. The three meat samples were cut into slices (about 3 cm × 2 cm × 0.5 cm) and then randomly distributed to the trained panelists. The panelists (25 men and 25 women, aged between 25 and 35) have undergone a selection and basic training to improve their ability before evaluating the taste. They were trained to know the products and the methodology and were to focus on the evaluation of the SPHSS, SPH and MRPH samples. Panelists were trained at least for two weeks. The training covered a first period to get familiar with the products, followed by a second period focused on the sample evaluation. During the training, descriptive terms were defined through panel discussion and references that showed intensities for each attribute were provided to the panel for calibration. Six sensory characteristics were set: sweetness (taste on the tongue refers to sucrose), umami (taste on the tongue refers to monosodium glutamate), bitterness (taste on the tongue refers to caffeine), sourness (taste on the tongue refers to citric acid), saltiness (taste on the tongue refers to sodium and chloride ions) and overall taste (the combination of different tastes).

The references were shown as follows: (1) 0.05 g caffeine/100 mL water = bitter 2, 0.08 g caffeine/100 mL water = bitter 5; (2) 2 g sucrose/100 mL water = sweet 2, 5 g sucrose/100 mL water = sweet 5; (3) 0.05 g citric/100 mL water = sour 2, 0.08 g citric/100 mL water = sour 5; (4) 0.5 g sodium chloride/100 mL in water = salty 5; and (5) 1 g monosodium glutamate/100 mL in water = umami 5 [15,16]. As the training went on, all members were able to recognize the six given descriptors and to use them consistently. Individual ratings for each attribute did not vary more than ±0.5 from the average panel score. Each member needed to grade the taste qualities from 1 to 5 points. The trained panelist performed a descriptive analysis of SPHSS, SPH and MRPH and evaluated their attributes. In order to prevent the effect of the different tastes among the three products, the panelists had to drink filtered water for mouth cleansing between samples.

## 3. Results and Discussion

### 3.1. H NMR Spectra of Extracts from SPHSS, SPH and MRPH

**Figure 1:**
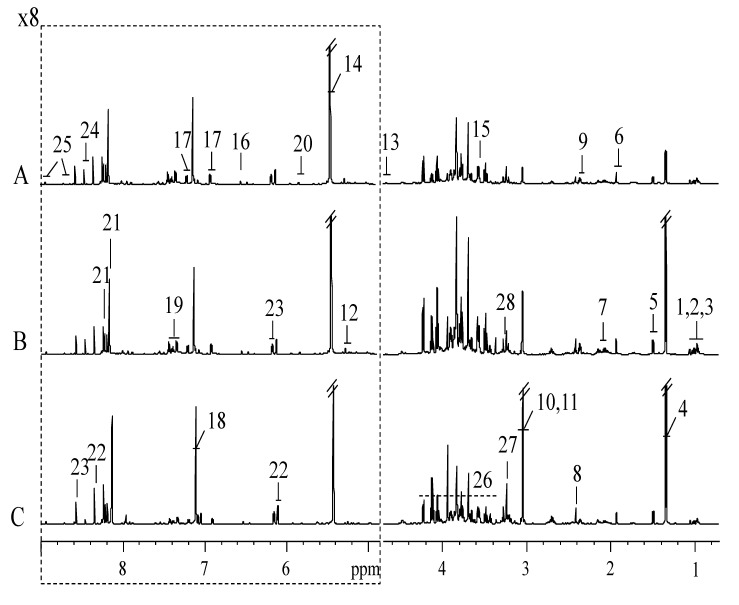
Representative 600 MHz ^1^H NMR spectra of stewed pork-hock in soy sauce (SPHSS) (**A**), stewed pork-hock (SPH) (**B**) and marinated and roasted pork-hock (MRPH) (**C**). The dotted region was vertically expanded eight times. Resonance assignments are given in Table 1. Keys: 1. isoleucine; 2. leucine; 3. valine; 4. lactate; 5. alanine; 6. acetate; 7. glutamate; 8. succinate; 9. pyroglutamate; 10. creatine; 11. creatinine; 12. α-glucose; 13. β-glucose; 14. sucrose; 15. glycine; 16. fumarate; 17. tyrosine; 18. histidine; 19. phenylalanine; 20. uracil; 21. hypoxanthine; 22. inosine; 23. 5′-IMP; 24. formate; 25. nicotinamide; 26. sugars and amino acids; 27. phosphorylcholine; 28. betaine.

Figure 1 showed the ^1^H NMR spectra of SPHSS, SPH and MRPH (A–C). According to both the ^1^H and ^13^C data placed in Table 1, all metabolites were identified with a series of 2D NMR experiments. The three meat products were dominated by 27 metabolites, including 10 amino acids (isoleucine, leucine, valine, alanine, glutamate, pyroglutamate, glycine, tyrosine, histidine and phenylalanine), three sugars (α-glucose, β-glucose and sucrose), six organic acids (lactate, acetate, succinate, creatine, fumarate and formate), four nucleotide metabolites (uracil, hypoxanthin, inosine and 5′-IMP), two alkaloids (phosphorylcholine and betaine) and two other metabolites (creatinine and nicotinamide) (Table 1). This is the first comprehensive research on the effect of the SD treatment of broth and its application in the processing of new meat products. According to the intensity and height of the peak spectrum, SPH contained higher contents of most metabolites compared with SPHSS and MRPH. This result was also corresponding to the OPLS-DA result and numerical value showed in Table 2.

### 3.2. The Analysis of Metabolites of SPHSS, SPH and MRPH

**Figure 2:**
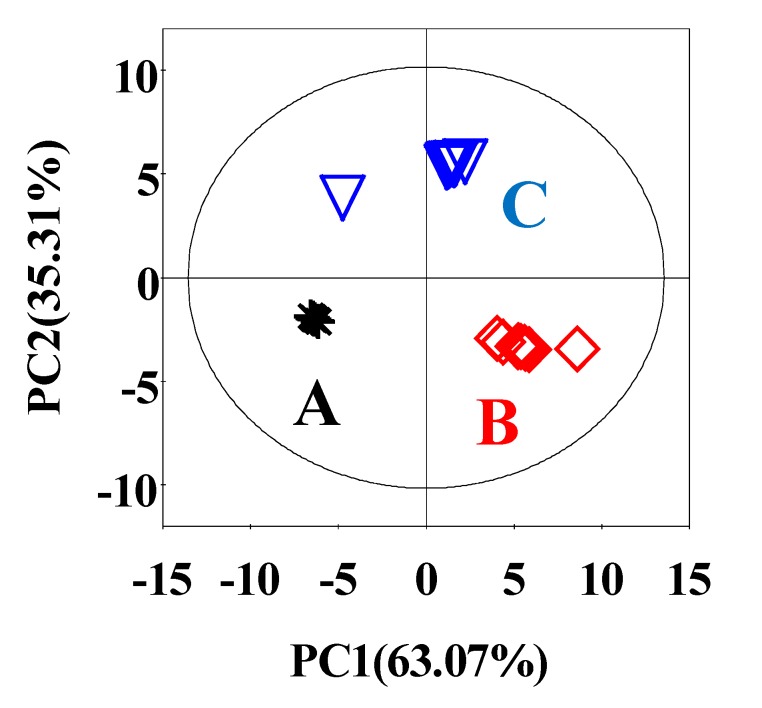
PCA score plots for SPHSS (**A**), SPH (**B**) and MRPH (**C**). PC1 and PC2 represent 63.07% and 35.31% of the total variance, respectively.

The PCA was conducted on the normalized NMR data. Figure 2 shows that PC1 and PC2 explained 63.07% and 35.31% of the total variation, respectively. It displays a metabolite profile variation. In PC1, differences were shown for the samples gained from SPHSS, SPH and MRPH (A–C). The three meat samples were located in three different parts of the PCA figure. SPHSS and SPH were located in the lower-half part of PCA. Their PC2 values were very similar, but PC1 values were greatly different. SPH contained the highest scores of the PC1 values, followed by MRPH and SPHSS. This result could be explained by the highest total FAAs and sugars in SPH.

**Figure 3:**
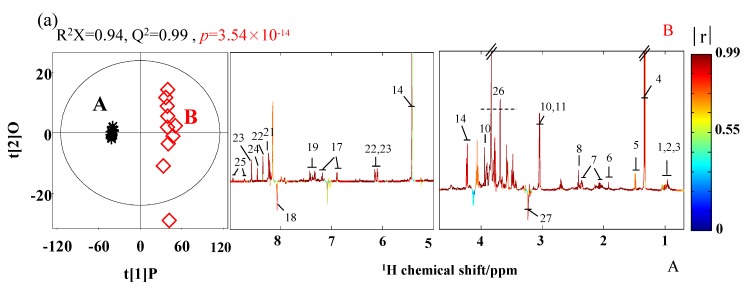

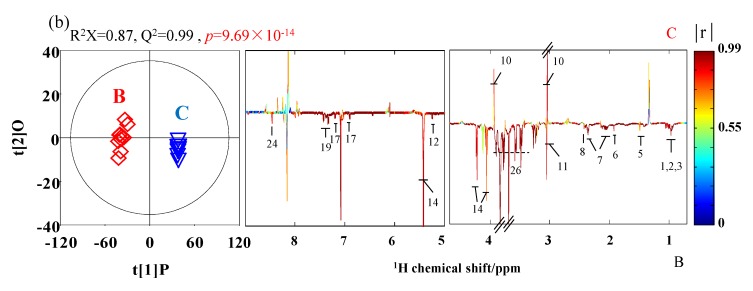
(**a**) OPLS-DA scores plots (left) and corresponding color-coded correlation coefficient loadings plots (right) from SPHSS (A) and SPH (B). (**b**) OPLS-DA scores plots (left) and corresponding color-coded correlation coefficient loadings plots (right) from SPH (B) and MRPH (C). The metabolite identification keys to the numbers are corresponded to Figure 1 and Table 1.

Figure 3 showed the pairwise comparative OPLS-DA scores plots and corresponding color-coded correlation coefficient loadings plots. The OPLS-DA from SPH and SPHSS (Figure 3a) as well as MRPH and SPH (Figure 3b) spectral data were performed, respectively. It represented an in-depth understanding of the metabolites and taste differences among the two comparative groups.

The quantitative values of R^2^X and Q^2^ on the OPLS-DA scores plots indicated that the quality of the two comparative groups were of reasonable quality. The *p* value represented a significant difference (*p* < 0.05) among the comparative groups. The color-coded correlation coefficient loadings plots (Figure 3, right) represented a significant difference. The peak spectrum color close to dark red represented the more marked contribution. On the contrary, the peak spectrum color close to cold blue represented the less marked contribution. In our study, the differences in metabolites with an absolute value of the correlation coefficient greater than 0.602 were regarded to be significant, which was equal to a significant level of discrimination against *p* < 0.05. The peak spectra of the metabolites presenting upward trends represent that their contents in the latter samples are higher than those in the former samples, while the spectra of metabolites presenting downward trends represent that their contents in the latter samples are lower than those in the former samples. The hotter the color of the variables is, the higher the significance between groups is.

The OPLS-DA results indicated that pork-hock processed by stewed treatment with reconstituted broth contains higher levels of metabolites. Meanwhile, the effect of the stewed method was different from the marinated coupled with roasting method on the sensory evaluation. In Figure 3a, compared with SPHSS, SPH samples contained higher contents of isoleucine, leucine, valine, lactate, alanine, acetate, glutamate, succinate, creatine, creatinine, sucrose, tyrosine, phenylalanine, hypoxanthine, inosine, 5′-IMP, formate and nicotinamide, but lower contents of histidine and phosphorylcholine. In Figure 3b, compared with SPH, MRPH samples contained a higher content of creatine, but lower contents of most metabolites, such as isoleucine, leucine, valine, alanine, acetate, glutamate, succinate, creatinine, α-glucose, sucrose, tyrosine, phenylalanine and formate. These results revealed a good matrix character and clear metabonomic difference between SPH and SPHSS, and also between MRPH and SPH.

Table 2 (list of metabolites) showed the coefficients for detected metabolites to explain the significance of their contributions. The B/A group showed a significant difference (|r| > 0.602) in all detected metabolic profiles, but not including formate, α-glucose and uracil. The C/B group showed a significant difference (*p* < 0.05) in all detected metabolic profiles (|r| > 0.602), but not including formate, uracil, inosine and nicotinamide. The result of the two comparative groups in Table 2 (list of metabolites) highly corresponded to the previous OPLS-DA results. To get more information about the difference of metabolites in SPHSS and SPH and in MRPH and SPH, the absolute quantification of the identified metabolites is shown in Table 2 (list of coefficients). The metabolite contents were calculated through equating the integrals of the NMR signal (least overlapping ones) in relation to that of the internal reference (TSP) with a known concentration.

Table 2 (list of coefficients) shows the metabolite content with SPH containing the highest total content of total FAAs (10.42 mg/g) compared with that in SPHSS (8.77 mg/g) and MRPH (5.17 mg/g); this could be caused by the migration of the original FAAs from the meat into the broth or by the degradation of the proteins and small peptides in soy sauce [17,18]. In addition, the ultra-fine powder could also promote the permeability characteristics of the meat under the heating conditions. Amino acids can be ascribed with different taste characteristics in meat [19]. Alanine and glycine are related to sweetness. Valine, tyrosine, isoleucine, leucine and phenylalanine have a relatively bitter taste. Glutamate presented an umami taste. In our study, the contents of isoleucine, alanine, glutamate, pyroglutamate and histidine were higher than the other FAAs. They could be the main FAAs that dominated the taste of the three products. Glutamate accounted for approximately 20% of the total FAAs, and was the major amino acid contributing to the intensity of the umami taste [20]. Glutamate could be induced by monosodium glutamate (MSG), which presents an umami taste and widely exists in soy sauces [18]. An umami taste produced a palatable taste and perception of satisfaction. Meanwhile, pyroglutamate imparts an umami taste with a similar activity to glutamate [21]. The content of glutamate in SPHSS (3.19 mg/g) was higher than in SPH (2.22 mg/g) and MRPH (1.01 mg/g). This result corresponded to the sensory evaluation results: The score of umami in SPH was not the highest among the three samples; this could be due to the weakened effect of the umami taste when drying the broth under a high temperature condition.

According to Pérezpalacios et al. [22], alanine and glycine have been identified to be the major amino acids in chicken soup. Glutamate, histidine and alanine were the most abundant amino acid in beef soups. These results highly corresponded to our results that the contents of glutamate, histidine, alanine and glycine were keep at high levels in SPH and MRPH. Kawai et al. [23] also found that the interaction of IMP combined with glycine and alanine could strongly enhance the effect of the umami taste. Isoleucine and histidine, as bitter FAAs, keep higher levels compared with other FAAs. The two FAAs kept the highest levels in the SPH samples (4.78 mg/g) compared with that in the SPHSS (3.88 mg/g) and MRPH (2.40 mg/g) samples. Paul A.S Breslin [24] found that bitter FAAs can further improve the umami taste of a mixture solution of glutamic acid. Therefore, the pork-hock processed by reconstituted broth present a more delicate flavor compared with traditional SPHSS.

As for the organic acids, fumarate and formate showed extremely low contents in the three products, which could be neglected regarding the impacts on taste. The contents of lactate and creatine presented higher trends in MRPH compared with SPH. Lactate is a mild acid that can lower the pH value in living animals and present a sour taste in cooked beef loins [25,26]. Although there were high levels of bitter FAAs and sour organics in the three samples, the bitterness and sourness were not significant according to results of the sensory evaluation. We speculate that the organic acid combined with other metabolites formed a new flavor, thus promoting the meat taste. Creatine plays an important role in muscle energy metabolism, which enhanced the characteristic flavor of thickness and mouthfulness [27,28,29]. In our research, the content of creatine is next to the content of lactate in the three products. The content of creatine in the MRPH (2.71 ± 0.31 mg/g) was highest and was nearly four times greater than that in the SPHSS (0.75 ± 0.10 mg/g). The creatine content in the SPH (1.93 ± 0.09 mg/g) was between that of the MRPH and SPHSS. Creatine is transformed into creatinine under heating conditions [30]. The SPH and SPHSS samples were under the same heating conditions but presented significantly different (*p* < 0.05) creatine contents; the result indicated that the reconstituted broth could improve the content of creatine. Compared to SPH, the highest content of creatine in MRPH could be due to the longer marinating time (12 h) than the stewed time (both are 90 min).

Sucrose dominated a great proportion of all metabolites in the three samples, probably due to the large addition of sugars in the stewing process. Sucrose provides a sweet taste, which could promote the meat taste. The content of sucrose is extremely high in the SPH (15.81 ± 0.68 mg/g) samples and more than double compared with that in SPHSS (7.21 ± 0.10 mg/g) and MRPH (6.36 ± 0.74 mg/g). We concluded that the extremely high level of sucrose in SPH may be caused by the large addition of sugars, which remains in the broth after stewing.

The nucleic acids and their derivatives were in relatively low concentrations compared with other kinds of metabolites in the three samples. The contents of uracil in the three samples were extremely low and the effect of uracil on taste could be neglected. The contents of 5′-IMP in the SPH (0.52 ± 0.02 mg/g) and MRPH (0.59 ± 0.07 mg/g) samples were nearly similar and both higher than that in the SPHSS (0.05 ± 0.00 mg/g) samples. 5′-IMP was important in meat flavor perception, as it holds umami taste characteristic [31]. 5′-IMP, as a flavor enhancer, was found to contribute to the sensory attributes of the brothy and meaty taste. Hypoxanthine, as the degradation product of 5′-IMP, was related to the bitter taste [32]. Hypoxanthine and inosine are the degradation compounds that are gradually transformed by 5′-IMP in meat [33]. The contents of the two compounds were significantly different (*p* < 0.05) in the three samples. According to Meelis et al. [32], the degradation of 5′-IMP proceed rapidly at a high temperature, which could explain the contents of hypoxanthine and inosine in MRPH being higher than that in SPHSS. The bitterness of hypoxanthine was not perceptible according to the sensory evaluation, which could be due to its low concentration or could be covered by other tastes, such as umami and sweetness. Although the nucleotide degradation products had relatively low levels in the three samples, they are still thought to be essential ingredients in meat flavor formation and development [32].

### 3.3. Sensory Evaluation of SPHSS, SPH and MRPH

**Figure 4:**
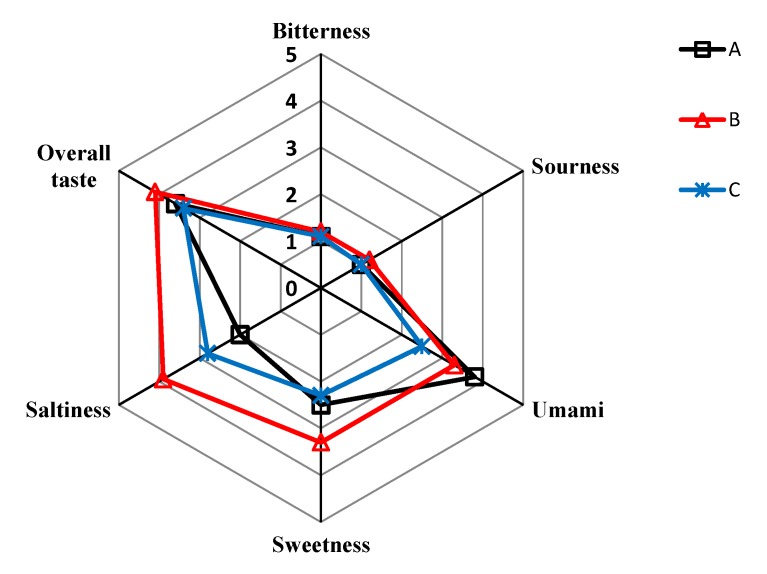
Sensory analysis result of SPHSS (A), SPH (B) and MRPH (C).

Figure 4 shows the sensory evaluation of the SPHSS (A), SPH (B) and MRPH (C) samples. It clearly shows that SPH contained the highest scores of sweetness and overall taste, followed by SPHSS and MRH. The overall taste scores could be related to the contents of total FAAs and sugars. The contents of total amino acids and sugars (Table 2) from high to low were SPH, SPHSS and MRPH, which could explain the highest overall taste score in SPH. The results indicated that the reconstituted broth could improve the intensity of the delightful taste in pork-hock with increasing the total FAAs and sugars. However, it did not enhance the umami taste. SPHSS contained a higher score of umami taste than that of SPH and MRPH. This result also corresponded to the contents of glutamate. We inferred that this could be caused by the decomposition of glutamate happening during SD treatment under a high temperature [34]. The scores of bitterness and sourness were at relative low levels, with both having nearly one point among the three products. We conclude that the SD treatment did not spoil the most taste-active compounds in the broth. In Figure 4, the line shape of the three meat products was significantly different (*p* < 0.05), which highly corresponds to the PCA result.

## 4. Conclusions

In SPH, the metabolite levels (alanine, lactate, glutamate, sucrose, betaine, creatine and succinate) were higher than that in SPHSS. SPH and SPHSS were under the same stewing conditions (80 °C for 90 min). However, the overall taste score and metabolite content of the SPH were also higher than for SPHSS and MRPH. High levels of total FAAs and total sugars in meat products could provide a better taste. We conclude that highly enriched, reconstituted broth significantly increases the metabolite levels in pork-hock. SPH and MRPH were under different processing conditions. In our results, the sensory score, the PC1 value and the height of the ^1^H NMR spectra were significantly higher than those in MRPH. According to these results, we conclude that the stewed processing method is more suitable for reconstituted broth compared to the marinated and roasted processing method. At the same time, the reconstituted broth (treated by spray drying) applied in possessing pork-hock was feasible and tasty.

## Figures and Tables

**Table 1:** NMR data of taste-active metabolites.

Key	Metabolites	Moieties	δ^1^ H (ppm) and Multiplicity^a^	δ^13^ C (ppm)
1	Isoleucine	αCH, βCH, γCH_2_, γ’CH_3_ δCH_3_	3.67(d), 1.98(m), 1.26(m), 1.45(m), 1.01(d), 0.94(t)	62.4, 38.6, 27.1, 17.7, 13.9
2	Leucine	αCH, βCH_2_, γCH, δCH_3_, δ’CH_3_	3.73(m), 1.73(m), 1.67(m), 0.98(d), 0.96(d)	56.3, 42.7, 26.8, 24.8, 23.8
3	Valine	αCH, βCH, γCH_3_, γ’CH_3_	3.62(d), 2.27(m), 0.99(d), 1.04(d)	63.2, 31.8, 19.3
4	Lactate	αCH, βCH_3_, COOH	4.11(q), 1.33(d)	71.2, 23.1, 185.3
5	Alanine	αCH, βCH_3_, COOH	3.78(q), 1.48(d)	53.3, 19.3, 178.7
6	Acetate	CH_3_, COOH	1.92(s)	26.3, 184.3
7	Glutamate	δCO, αCH, βCH_2_, γCH_2_, COOH	3.77(m), 2.12(m), 2.05(m), 2.36(dt)	184.2, 57.1, 29.8, 36.2, 177.7
8	Succinate	CH_2_	2.41(s)	36.9, 184.6
9	Pyroglutamate	αCH, βCH_2_, γCH_2_	4.19(dd), 2.52(m), 2.04(m), 2.41(m),	61.2, 28.3, 32.9
10	Creatine	CH_2_, NCH_3_, CNH, COOH	3.93(s), 3.04(s)	39.8, 56.8, 160.3, 177.7
11	Creatinine	CH_2_, NCH_3_, CNH, CO	4.06(s), 3.05(s)	59.1, 32.9, 172.1, 191.6
12	α-Glucose	C_1_H, C_2_H, C_3_H	5.24(d), 3.54(d)	95.0, 73.0
13	β-Glucose	C_1_H, C_2_H, C_3_H	4.65(d), 3.25(t), 3.50(dd)	98.6, 77.1, 78.7
14	Sucrose	G_1_H, G_2_H, G_3_H, G_4_H, G_5_H, G_6_H, F_1_H, F_2_, F_3_H, F_4_H, F_5_H, F_6_H	5.42(d), 3.58(dd), 3.77(t), 3.49(t), 3.82(q), 3.81(q), 3.69(s), 4.23(d), 4.06(t), 3.91(m), 3.83	94.7, 73.5, 75.0, 71.8, 74.9, 62.8, 64.0, 106.1, 79.2, 76.6, 83.7, 65.0
15	Glycine	αCH_2_, COOH	3.57(s)	44.5, 175.4
16	Fumarate	CH, COOH	6.53(s)	138.7, 177.5
17	Tyrosine	C_1_, Ring, C_2,6_H, Ring, C_3,5_H, Ring, C_4_, Ring	7.20(d), 6.90(d)	129.5, 133.6, 118.8, 157.5
18	Histidine	αCH, βCH, γC	8.06(s), 7.12(d)	136.8, 133.8, 119.7
19	Phenylalanine	C_1_, Ring, C_2,6_H, Ring, C_3,5_H, Ring, C_4_H, Ring	7.33(d), 7.43(t), 7.38(m)	137.9, 132.6, 132.0, 130.7
20	Uracil	C_3_H, C_4_H, C_1,_ C_2_	7.54(d), 5.81(d)	144.6, 103.4, 170.2, 156.1
21	Hypoxanthine	C_2_H, C_6_H	8.22(s), 8.20(s)	144.6, 147.8
22	Inosine	C_1_H, C_2_, C_3_, C_4_H, C_5_, C_1′_H, C_2′_H, C_3′_H, C_4′_H, C_5′_H	8.34(s), 8.23(s), 6.10(d), 4.79, 4.44(m), 4.28 (m), 3.91, 3.83	143.0, 127.2, 161.8, 149.1, 151.5, 91.2, 77.1, 73.3, 88.4, 63.9
23	5′-IMP	C_1_H, C_2_, C_3_, C_4_H, C_5_, C_1′_H, C_2′_H, C_3′_H, C_4′_H, C_5′_H	8.58(s), 8.24(s), 6.15(d), 4.81(#), 4.51(m), 4.38 (m), 4.06(#)	142.7, 126.7, 161.8, 149.1, 152.0, 90.2, 77.6, 73.5, 87.4, 66.7
24	Formate	CH	8.46(s)	174.2
25	Nicotinamide	C_2_H, C_4_H, C_5_H, C_6_H	8.94(dd), 8.27(dd), 7.60(dd), 8.72(dd)	150.1, 139.0, 127.0, 154.2
26	Sugars and amino acids	αCH resonances	3.3-3.9	
27	Phosphorylcholine	αCH_2_, βCH_2_, N-CH_3_	4.08(d), 3.55 (d), 3.23(s)	69.0, 56.2
28	Betaine	CH_3_, CH_2_, COOH	3.28(s), 3.93(s)	56.7, 68.9, 172.2

^a^ Multiplicity: s, singlet; d, doublet; t, triplet; q, quartet; dd, doublet of doublets; dt, doublet of triples; m, multiplet. #, the multiplicities were not determined.

**Table 2:** Coefficients from OPLS-DA and the contents of the taste-active metabolites.

Metabolite	Coefficient ^1^		Mean ± SD ^2^		
	B/A	C/B	A (mg/g)	B (mg/g)	C (mg/g)
Isoleucine	0.99	−0.97	1.87 ± 0.02b	2.95 ± 0.04a	1.80 ± 0.09 b
Leucine	0.99	−0.99	0.19 ± 0.00b	0.34 ± 0.02a	0.16 ± 0.02c
Valine	0.98	−0.99	0.13 ± 0.00b	0.26 ± 0.01a	0.13 ± 0.01b
Alanine	0.83	−0.92	0.23 ± 0.00c	0.50 ± 0.02a	0.37 ± 0.04b
Glutamate	0.99	−0.99	3.19 ± 0.02a	2.22 ± 0.10b	1.01 ± 0.02c
Pyroglutamate	0.99	−0.99	0.87 ± 0.01b	1.85 ± 0.08a	0.88 ± 0.05b
Glycine	/	/	/	/	/
Tyrosine	0.94	−0.95	0.11 ± 0.00b	0.18 ± 0.00a	0.09 ± 0.01b
Histidine	−0.81	−0.99	2.01 ± 0.27a	1.83 ± 0.15b	0.60 ± 0.01c
Phenylalanine	0.97	−0.99	0.17 ± 0.00b	0.29 ± 0.01a	0.13 ± 0.02c
Total FAAs			8.77	10.42	5.17
Lactate	0.99	0.95	0.88 ± 0.01c	3.81 ± 0.16b	4.49 ± 0.51a
Acetate	0.97	−0.97	0.10 ± 0.00b	0.17 ± 0.01a	0.10 ± 0.01b
Succinate	0.99	−0.95	0.14 ± 0.00c	0.37 ± 0.02a	0.27 ± 0.03b
Creatine	0.99	0.99	0.75 ± 0.10c	1.93 ± 0.09b	2.71 ± 0.31a
Fumarate	0.91	−0.86	0.01 ± 0.00b	0.02 ± 0.00a	0.01 ± 0.00b
Formate	−	−	0.01 ± 0.00b	0.04 ± 0.00a	0.01 ± 0.00b
Total organic acids			1.88	6.34	7.59
α-Glucose	−	−0.98	0.19 ± 0.00a	0.21 ± 0.03a	0.07 ± 0.01b
β-Glucose	0.96	−0.99	0.31 ± 0.01b	0.46 ± 0.03a	0.15 ± 0.01c
Sucrose	0.98	−0.98	7.21 ± 0.10b	15.81 ± 0.68a	6.36 ± 0.74c
Total sugars			7.71	16.48	6.58
Uracil	−	−	0.04 ± 0.00b	0.07 ± 0.00a	0.04 ± 0.00b
Hypoxanthine	0.98	−0.89	0.13 ± 0.00c	0.27 ± 0.01a	0.19 ± 0.02b
Inosine	0.99	−	0.14 ± 0.00c	0.51 ± 0.02b	0.58 ± 0.07a
5′-IMP	0.99	0.87	0.05 ± 0.00c	0.52 ± 0.02b	0.59 ± 0.07a
Total nucleic acids and their derivatives			0.36	1.37	1.4
Creatinine	0.81	−0.92	1.14 ± 0.46b	2.25 ± 0.43a	1.03 ± 0.02c
Nicotinamide	0.96	−	0.01 ± 0.00b	0.05 ± 0.00a	0.04 ± 0.00a
Phosphorylcholine	−0.87	−0.98	0.96 ± 0.02a	0.67 ± 0.02b	0.34 ± 0.01c
Betaine	0.99	0.99	0.67 ± 0.01c	1.73 ± 0.08b	2.51 ± 0.04a

^a–c^ Different letters mean significant difference among different samples (*p* < 0.05). A = SPHSS; B = SPH; C = MRPH. ^1^ The coefficients from OPLS-DA results, positive and negative signs indicate positive and negative correlation in the concentrations. The coefficient of 0.602 was used as the cut-off value for the significant difference (*p* < 0.05). “−” represents the absolute value of the coefficient is < 0.602. “/” represents the absolute concentration was not calculated due to signal overlapping. ^2^ The absolute concentration and standard deviation (Mean ± SD) were obtained from 10 parallel samples.
